# Zipper-interacting protein kinase promotes epithelial-mesenchymal transition, invasion and metastasis through AKT and NF-κB signaling and is associated with metastasis and poor prognosis in gastric cancer patients

**DOI:** 10.18632/oncotarget.3200

**Published:** 2015-03-21

**Authors:** Jian Li, Zhijuan Deng, Zhu Wang, Dong Wang, Longjuan Zhang, Qiao Su, Yingrong Lai, Bin Li, Zexing Luo, Xu Chen, Yu Chen, Xiaohui Huang, Jieyi Ma, Wenjian Wang, Jiong Bi, Xinyuan Guan

**Affiliations:** ^1^ Laboratory of General Surgery, The First Affiliated Hospital, Sun Yat-Sen University, Guangzhou 510080, China; ^2^ State Key Laboratory of Oncology in South China, Sun Yat-Sen University Cancer Center, Guangzhou 510060, China; ^3^ Department of Clinical Laboratory, Sun Yat-Sen Memorial Hospital, Sun Yat-Sen University, Guangzhou 510120, China; ^4^ Department of Medical Ultrasonics Institute of Diagnostic and Interventional Ultrasound, The First Affiliated Hospital, Sun Yat-Sen University, Guangzhou 510080, China; ^5^ Department of Clinical Laboratory, The First Affiliated Hospital, Sun Yat-Sen University, Guangzhou 510080, China; ^6^ Department of Pathology, The First Affiliated Hospital, Sun Yat-Sen University, Guangzhou 510080, China; ^7^ School of Public Health, Sun Yat-Sen University, Guangzhou 510074, China; ^8^ Animal Center, The First Affiliated Hospital, Sun Yat-Sen University, Guangzhou 510080, China

**Keywords:** zipper-interacting protein kinase, epithelial-mesenchymal transition, gastric cancer, metastasis, AKT/IκB/NF-κB pathway

## Abstract

Zipper-interacting Protein Kinase (ZIPK) belongs to the death-associated protein kinase family. ZIPK has been characterized as a tumor suppressor in various tumors, including gastric cancer. On the other hand, ZIPK also promotes cell survival. In this study, both *in vitro* and *in vivo* assays indicated that ZIPK promoted cell growth, proliferation, migration, invasion, tumor formation and metastasis in nude mice. ZIPK induced epithelial-mesenchymal transition (EMT) with increasing expression of β-catenin, mesenchymal markers, Snail and Slug, and with decreasing expression of E-cadherin. Furthermore, ZIPK activated the AKT/IκB/NF-κB pathway, which can promote EMT and metastasis. Additionally, ZIPK expression was detected in human primary gastric cancer and their matched metastatic lymph node samples by immunohistochemistry. Increased expression of ZIPK in lymph node metastases was significantly associated with stage VI and abdominal organ invasion. Survival analysis revealed that patients with increased ZIPK expression in metastatic lymph nodes had poor disease-specific survival. Taken together, our study reveals that ZIPK is a pro-oncogenic factor, which promotes cancer metastasis.

## INTRODUCTION

Gastric cancer (GC) is the second most common cause of cancer-related death in the world and 42% of the cases occur in China [1]. Although the surgical approaches and adjuvant chemotherapy have been improved, the survival rate of GC patients at advanced stages remains low. The treatment failure may reflect the poorly understood biology of gastric cancer cells and their underlying signal transduction pathways. Accumulating evidence has suggested that the deregulation of cell death and survival pathways may promote tumor development and progression, with potentially devastating consequences for cancer patients [2]. Therefore, it is imperative to investigate the molecular mechanisms of gastric cancer to improve therapeutic approaches and patient outcomes.

Zipper-interacting Protein Kinase (ZIPK), or DAPK3, belongs to the death-associated protein kinase family which also includes DAPK1 and DAPK2. As their name imply, the three kinases share significant homology within their common N-terminal kinase domain and share some functions such as caspase-dependent and -independent cell death induction. ZIPK colocalizes with Par4 at actin filaments to cause the formation of thick actin bundles and apoptotic events [3]. ZIPK also induces the diphosphorylation of the regulatory light chain of myosin II in non-muscle cells and gives rise to apoptotic morphological changes [4]. Moreover, ZIPK is a downstream target of DAPK1 and may potentiate DAPK1-mediated apoptosis [5]. On the other hand, ZIPK has a critical role in the regulation of starvation-induced autophagy and Atg9-mediated autophagosome formation through the myosin II motor protein [6]. ZIPK has been linked to several cell death–related signaling pathways. In accordance with its cell death promoting activity, some evidences suggest that ZIPK functions as a tumor suppressor. ZIPK mRNA expression was reportedly decreased in skin squamous cell carcinoma [7]. Our previous study showed that decreased expression of ZIPK was detected in 111/162 primary gastric carcinomas, which was significantly associated with invasion, metastasis and poor prognosis [8]. Furthermore, somatic ZIPK mutations have been discovered in colon cancer, ovarian cancer and lung cancer. Such mutations block kinase activity and promote cancer cell survival [9].

However, the DAPK family exhibits anti-apoptotic activity under certain conditions. Silencing of DAPK by siRNA could result in apoptosis [10]. DAPK1 positively regulates mTORC1 in response to growth factor activation by binding to TSC2 and regulating its phosphorylation. The novel link between DAPK1 and mTORC1 signaling pathways has suggested that DAPK1 plays a pivotal role in cell survival [11]. Recently, ZIPK has been described as a novel transcriptional co-activator of androgen receptor (AR). ZIPK may regulate AR anti-apoptotic and proliferative functions and provide a growth advantage to prostate cancer cells [12]. Until now, the nature of ZIPK in gastric cancer pathogenesis is unclear. In the current study, we used both *in vitro* and *in vivo* assays to characterize ZIPK function in gastric cancer cell lines. We found that ZIPK activates AKT/IκB/NF-κB pathway which could promote cancer cell epithelial-mesenchymal transition and metastasis. Increased expression of ZIPK merely in human metastatic lymph nodes compared to the expression of ZIPK in their primary GCs is associated with abdominal organ metastases and patient's poor survival.

## RESULTS

### Overexpression of ZIPK increases cell growth and proliferation

**Figure 1:**
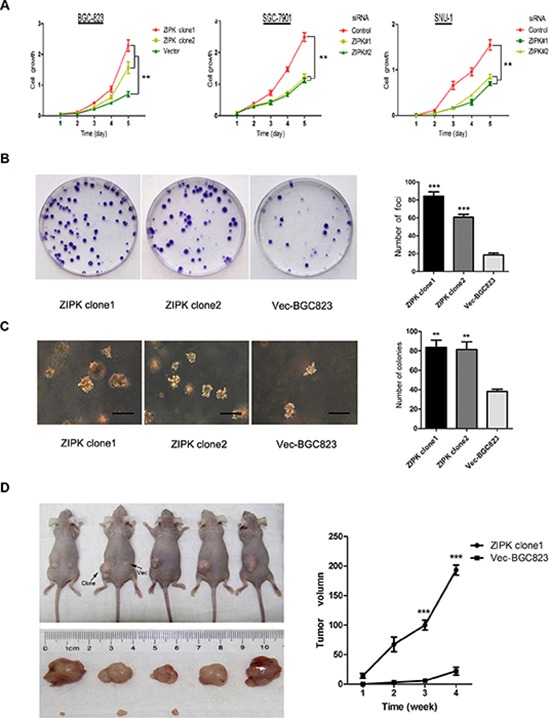
ZIPK increases tumor growth and proliferation *in vitro* and *in vivo* **(A)** XTT assay showed that over-expression of ZIPK increased cell proliferation, and ablation of endogenous ZIPK inhibited cell proliferation. The results were expressed as the mean ± SD of three independent experiments (**indicates *P* < 0.01 in independent Student's *t*-test). **(B)** Foci formation assay was used to compare the frequency of foci formation between ZIPK- and vector-transfected cells. Quantitative analyses of foci numbers was shown in the right panel. Values was reflected as the mean ± SD of at least three independent experiments (***indicates *P* < 0.001, independent Student's *t*-test). **(C)** Ability to form colony in soft agar increased significantly in ZIPK-transfected cells compared with vector cells (**P < 0.01, independent Student's *t*-test. Scale bars: 100 μm). **(D)** ZIPK- and empty vector-transfected cells were injected into the left and right dorsal area of the nude mice, respectively. ZIPK mediated tumor growth *in vivo*. The average tumor volume was expressed as the mean ± SD of five inoculated sites for each group (***P < 0.001).

To characterize the biological effect of ZIPK on cell growth and proliferation in gastric cancer cell lines, ZIPK was stably transfected into BGC-823 cells (ZIPK clone1 or ZIPK clone2). Empty vector–transfected cells were used as control (Vec-BGC823). Meanwhile, ZIPK in SGC-7901 and SNU-1 cells were silenced by siRNAs. Ectopic overexpression and decreased expression of ZIPK were determined by RT-PCR and Western blotting (Supplementary Figure 1). XTT assays showed that ZIPK overexpression markedly increased BGC-823 cell proliferation. Conversely, silencing ZIPK inhibited cell proliferation in SGC-7901 and SNU-1 cells (*P* < 0.01 Figure 1A). Anchorage-dependent foci formation and anchorage-independent colony formation in soft agar yielded a higher number and larger colonies (*P* < 0.01) in the ZIPK-transfected cells compared to the control cells (Figure 1B, 1C). The tumorigenic potential of ZIPK was also evaluated by xenograft tumor formation in athymic nude mice. Subcutaneous visible tumors were observed in the left flank (ZIPK clone1) in all 5 tested animals on day 7 after injection. However, visible tumor in the right flank (Vec-BGC823) was only observed in 2 nude mice on day 14. Xenograft tumor growth curve indicated that tumors induced by ZIPK-transfeced cells grew much more rapidly than tumors induced by Vec-BGC823 cells (*P* < 0.001; Figure 1D). On day 28 after injection, tested mice were sacrificed and the tumors were excised for further analysis. The average volume of tumors induced by ZIPK clone cells (186 ± 48.8 mm^3^) was significantly increased compared with tumors induced by Vec-BGC823 cells (8.8 ± 10.5 mm^3^, *P* < 0.001; Figure 1D).

### ZIPK promotes cell invasion *in vitro* and tumor metastasis *in vivo*

**Figure 2:**
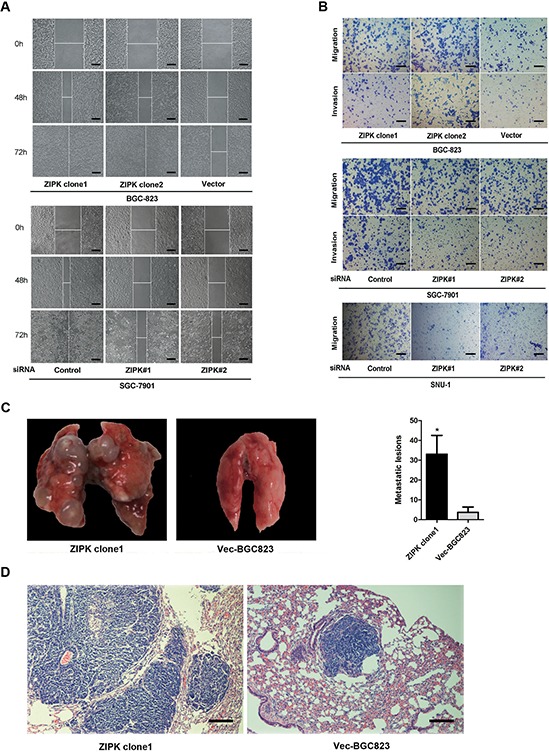
ZIPK promotes cell invasion and gastric cancer metastasis **(A)** Wound-healing assay showed that over-expression of ZIPK promoted cell migration. Silencing ZIPK inhibited cell migration (Scale bars: 100 μm). Representative images were taken at 0 h, 48 h and 72 h after scratching. **(B)** Transwell assay indicated that over-expression of ZIPK promoted cell migration and invasion. Inversely, ZIPK ablation repressed cell migration and invasion (Scale bars: 100 μm). **(C)** The effects of ZIPK on tumor metastasis *in vivo* were evaluated by tail vein injections of cells in nude mice. Representative images of lungs derived from nude mice injected with ZIPK- or empty vector-transfected BGC823 cells were shown in the left panel. Number of visible surface metastatic lesions was indicated in the right panel (*indicates *P* < 0.05, independent Student's *t*-test). **(D)** Lung metastases in the mice were confirmed by H&E staining (Scale bars: 100 μm).

After confirming the tumorigenic ability of ZIPK, we investigated the roles of ZIPK in cell migration, invasion and tumor metastasis. Wound healing assay showed that ZIPK-transfected cells obtained quicker closure of the scratched “wound” and silencing ZIPK resulted in a significant delay of scratch area closure (Figure 2A). In addition, we further measured cell migration and invasion using Transwell assays in ZIPK-transfected, ZIPK-silenced and their respective control cells. ZIPK overexpression significantly increased cell migration and invasiveness in BGC-823 cell line. Knockdown of ZIPK repressed cell migration and invasion in SGC-7901 and SNU-1 cells (*P* < 0.001; Figure 2B). To evaluate the *in vivo* effects of ZIPK on tumor metastasis, two groups of 5 mice each were injected intravenously in the tail vein with ZIPK-transfected cells or Vec-BGC823, respectively. After 8 weeks, the mice were sacrificed, and the metastatic nodules at the lung surfaces were counted. A significantly larger number of metastatic nodules were induced at the surface of the lungs of mice injected with the ZIPK-transfected cells than those with the Vec-BGC823 cells (*P* < 0.001, Figure 2C). Hematoxylin and eosin (H&E) staining confirmed that the nodules on the surfaces of mice lungs were metastatic tumors. Histological analyses further revealed that ZIPK promoted metastasis of BGC-823 cell line (Figure 2D).

### ZIPK induces epithelial-mesenchymal transition in gastric cancer cells

**Figure 3:**
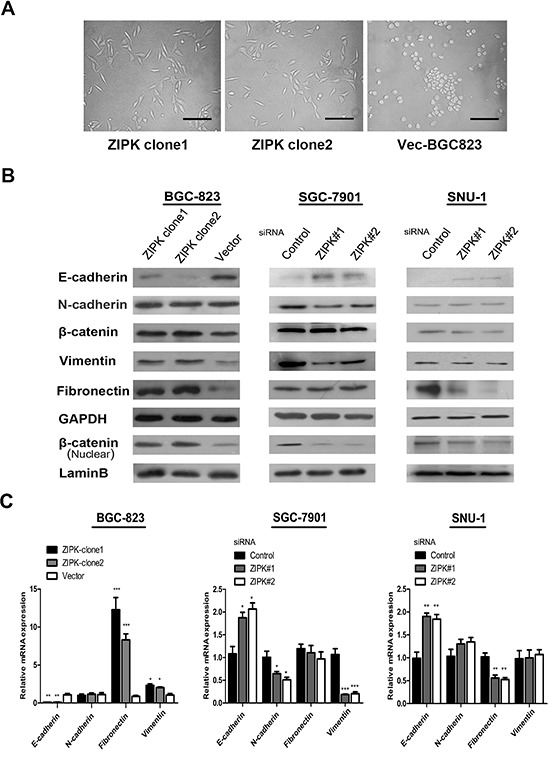
ZIPK induces epithelial-mesenchymal transition **(A)** Effect of ZIPK over-expression on cell morphology evaluated by phase-contrast microscopy (Scale bars: 100 μm). **(B)** Expression of epithelial cell marker (E-cadherin), mesenchymal cell markers (vimentin, fironectin and N-cadherin) and β-catenin were examined by Western blot analysis in ZIPK-overexpressed, ZIPK-silenced and their respective control cells. GAPDH was used as a loading control. Intensities of bands were analyzed by the Macintosh densitometry program Image J (NIH, Bethesda, MD). **(C)** Relative expressions of E-cadherin, N-cadherin, vimentin, fibronectin, β-catenin were detected by qPCR in ZIPK-overexpressed, ZIPK-silenced and their respective control cells.

As shown in Figure 3A, overexpression of ZIPK led to altered morphological characteristics of epithelial-mesenchymal transition (EMT), identified by a scattered distribution of cells and spindle or star-like morphology of the cells in culture. Since the *in vitro* and *in vivo* experiments showed that ZIPK could play a pivotal role in gastric cancer cell metastasis, we next asked whether ZIPK affect on cell biological programs that initiate metastasis cascades. EMT has been recognized as a critical event in tumor metastasis. To validate whether ZIPK enhances gastric cancer metastasis via inducing EMT, the expression levels of EMT markers were detected by Western blot and qRT-PCR in ZIPK-transfected, ZIPK-silenced and their respective control cells. We found that the epithelial marker, E-cadherin was dramatically down-regulated in the ZIPK-transfected cells, but mesenchymal markers such as vimentin and fibronectin were strongly up-regulated in ZIPK transfectcted BGC-823 cells. Moreover, overexpression of ZIPK increased β-catenin and its nuclear translocation. As expected, opposite expression profiles of these proteins were detected in ZIPK-silenced cells (Figure 3B, 3C). Increased nuclear accumulation of β-catenin is a known transcription factor that regulates the activity of TCF/LEF family to facilitate EMT and metastasis [13]. Collectively, these data suggest that ZIPK might be a positive mediator of EMT and metastasis in gastric cancer cells.

### ZIPK activates AKT/IκB/NF-kB signaling to induce EMT

**Figure 4:**
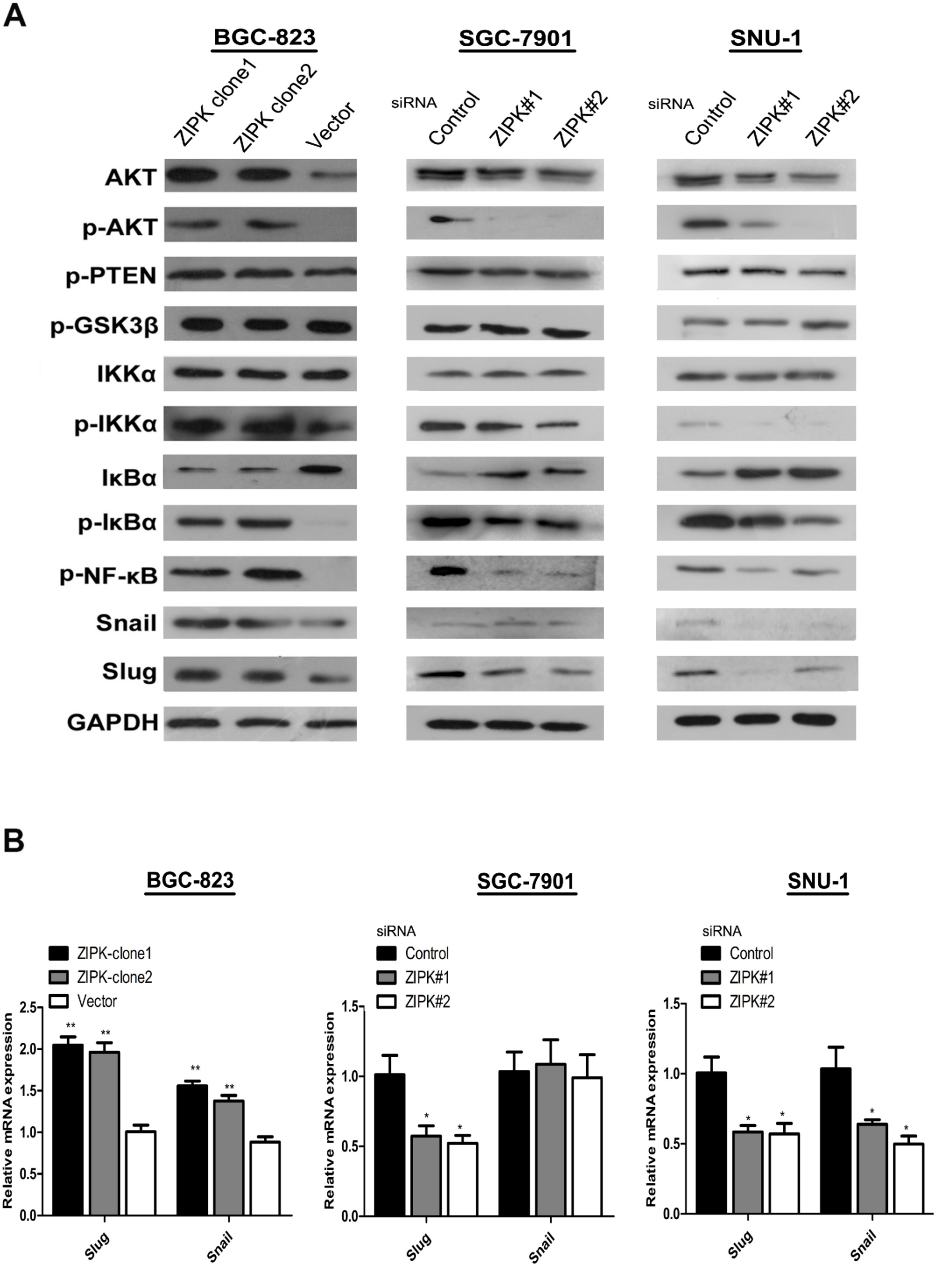
ZIPK activates AKT/IκBα/NF-κB pathway to induce EMT **(A)** Western blot analysis showed the protein levels of AKT, pAKT, pPTEN, pGSK3β, IKKα, p-IKKα, IκBα, p-IκBα, p-NF-κB, Snail and Slug in ZIPK-overexpressed, ZIPK-silenced and their respective control cells. **(B)** Relative mRNA expression of Snail and Slug were detected by qPCR in ZIPK-overexpressed, ZIPK-silenced and their respective control cells.

**Figure 5:**
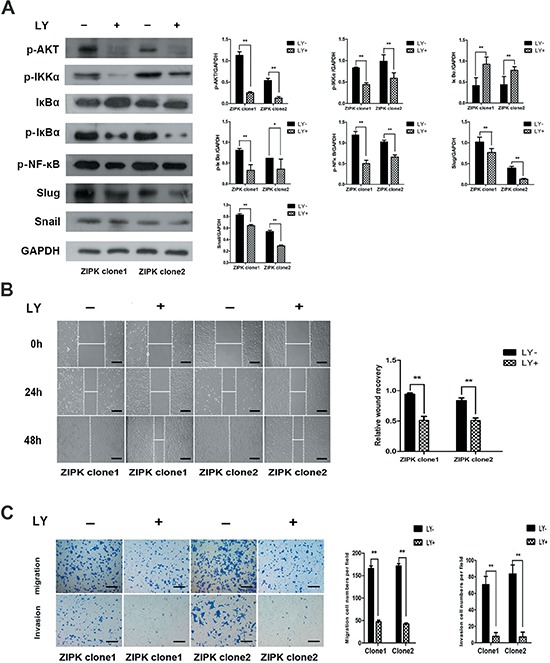
The AKT inhibitor LY294002 decreases ZIPK-induced cell migration and invasion **(A)** ZIPK-overexpressed cells were treated with LY294002 (20 μM) or DMSO for 2 h, the expressions of pAKT, IKKα, p-IKKα, IκBα, p-IκBα, p-NF-κB, Snail and Slug were detected by Western blotting. **(B)** Wound-healing assay showed that LY294002 strongly inhibited cell migration (**indicates *P* < 0.01, independent Student's *t*-test, scale bars: 100). **(C)** Transwell assay indicated that LY294002 reduced ZIPK-induced cell migration and invasion (**indicates *P* < 0.01, independent Student's *t*-test, scale bars: 100).

To further explore the molecular mechanisms responsible for ZIPK mediated EMT, we focused on AKT signaling because activation of AKT is a central feature of EMT and ZIPK has been demonstrated to increase AKT activation in spontaneously hypertensive rats [14]. We examined the expression levels of phosphorylated AKT and AKT by Western blotting. ZIPK-transfected BGC823 cells displayed high levels of phosphorylated AKT than did empty vector-transfected cells (Figure 4A). AKT does not directly regulate gene transcription, but it phosphorylates many substrates that activate downstream EMT-related gene. GSK-3β has been characterized as a main kinase responsible for protein stability of Snail [15]. However, in this study, we did not detect any change in GSK-3β activity between ZIPK-transfected and vector-transfected BGC823 cells (Figure 4A). The AKT/IκB pathway has also been reported to strongly regulate NF-κB expression and activation [16]. Moreover, NF-κB can modulate EMT phenotype via inducing Snail and Slug expression [17, 18]. Therefore, we examined the expression of AKT downstream targets, NF-κB, Snail and Slug by Western blot analysis. Indeed, we found that p-IKKα, p-IκBα, NF-κB, Snail and Slug were significantly up-regulated and that IκBα was down-regulated in ZIPK-transfected cells (Figure 4A). The Slug mRNA levels were higher and the Snail mRNA levels were slightly increased in the ZIPK-transfected cells (Figure 4B). Knockdown of ZIPK dramatically decreased p-IKKα, p-IκBα and NF-κB expression and increased IκBα level. Silencing ZIPK inhibited slug expression in SGC7901 cells and decreased both slug and snail expression in SNU-1 cells (Figure 4A, 4B). These data suggest that ZIPK overexpression enhances AKT/IκB/NF-κB signaling. To verify the crucial role of AKT signaling pathway in ZIPK induced EMT, we treated cells with the specific PI3K/AKT antagonist, LY294002. After treating ZIPK-transfected cells with LY294002 for 2 h, we examined pAKT, AKT, p-IKKα, p-IκBα, IκBα, NF-κB, Snail, and Slug expression. The results showed that AKT inactivation dramatically suppressed p-IKKα, p-IκBα, NF-κB, Snail and Slug expression and restored IκBα expression (Figure 5A). In addition, wound healing assay and Transwell assay demonstrated that LY294002 could significantly decrease cell migration and invasion abilities in ZIPK-transfected cells (Figure 5B, 5C). Since PTEN is a major negative regulator of the PI3K/AKT signaling pathway, we investigated whether ZIPK has a effect on PTEN protein stability. Phosphorylation of PTEN (ser380) was detected by Western blotting. We found that ZIPK did not seem to regulate PTEN stability (Figure 4A).

### ZIPK increases stemness of gastric cancer cells

**Figure 6:**
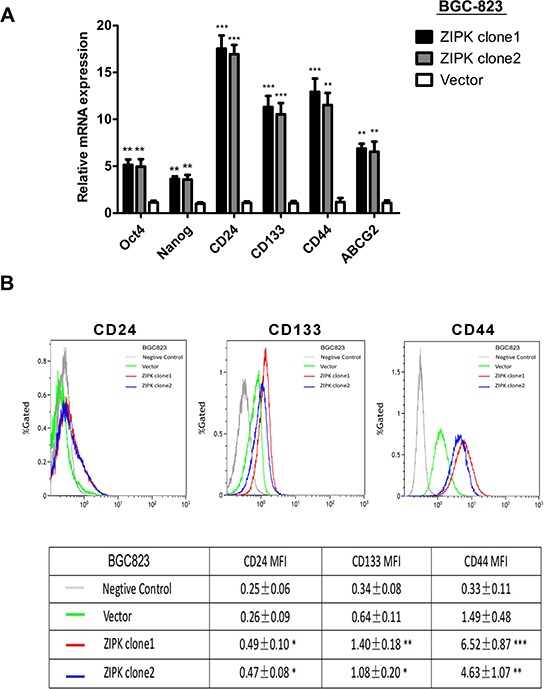
ZIPK enhances stemness of gastric cancer cells **(A)** Quantitative real-time PCR analysis showed the elevated expression of Oct-4, Nanog, ABCG2, CD44, CD24 and CD133 in ZIPK-transfected cells compared with control cells (**indicates *P* < 0.01; ***indicates *P* < 0.001, independent Student's *t*-test). **(B)** Flow cytometry analysis showed the higher mean flourscence indensity of FITC-CD44, PE-CD24, and PE-CD133 in ZIPK-transfected cells (**indicates *P* < 0.01, independent Student's *t*-test).

There is emerging evidence that the EMT process may give rise to cancer stem cells (CSCs) or cells with stem cell-like properties. We further examined if ZIPK could increase stemness of gastric cancer cells by determining expression levels of stemness-associated genes through qRT-PCR. The results showed that stemness genes (Nanog and Oct-4), multiple drug-resistant transporter gene (ABCG2) and surface antigens associated with cancer stem cells (CD24, CD44 and CD133) were upregulated in ZIPK-transfected cells compared with control cells (Figure 6A). Flow cytometry assay showed that the expression level of CD44, CD24 and CD133 was high in ZIPK-transfected cells (Figure 6B).

### Clinical association of ZIPK expression in primary gastric cancers and the matched metastatic lymph nodes

**Figure 7:**
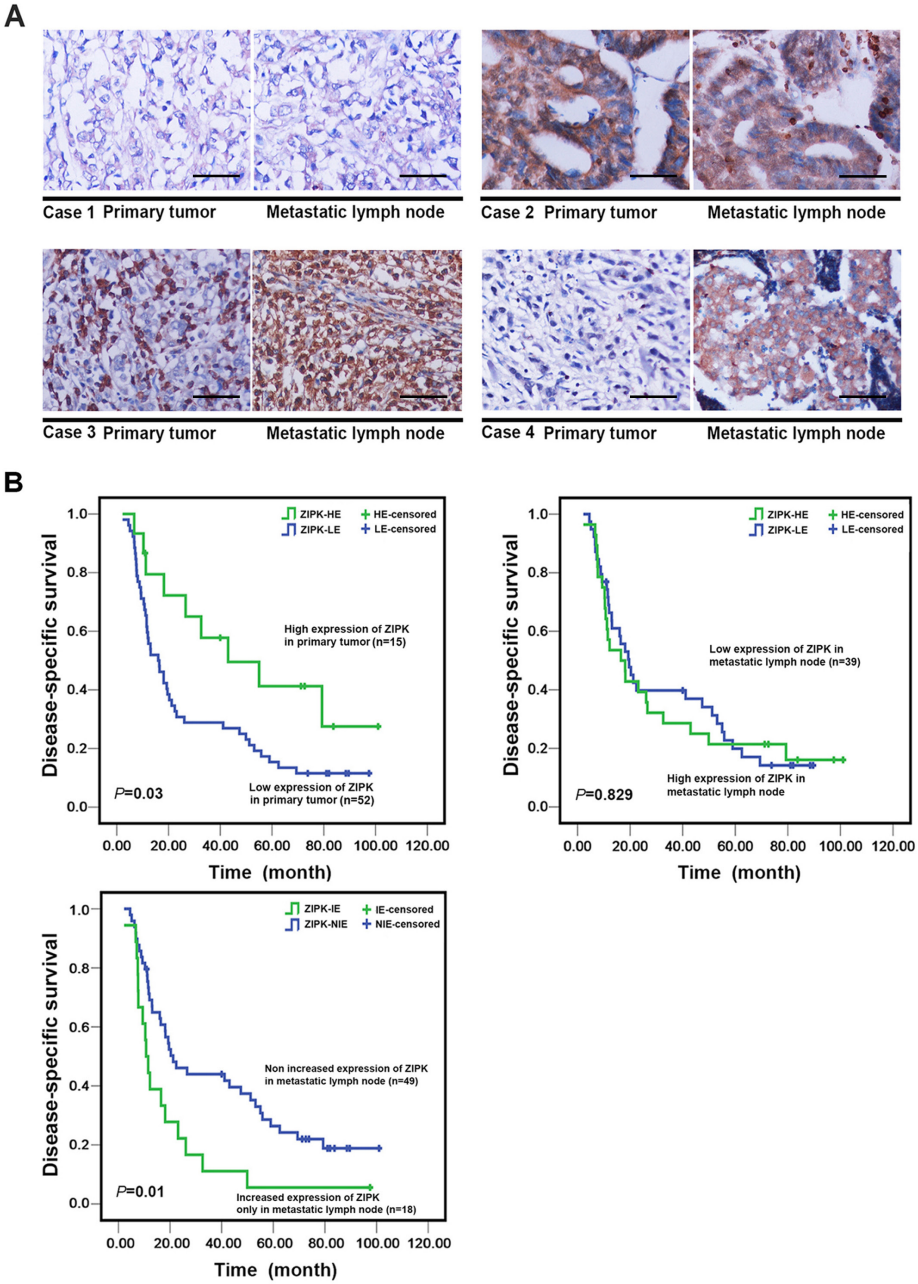
The prognostic significance of ZIPK expression in primary GCs and matched metastatic lymph nodes **(A)** Immunohistochemical analysis of ZIPK expression in primary cancer tissues and matched lymph node metastases (200 × magnification, Scale bars: 100). Representative images in the upper panel illustrated that the levels of ZIPK expression were not different between primary and metastatic cancers. Images in the lower panel showed increased ZIPK expression in lymph node metastases compared to their primary tumors. **(B)** Kaplan–Meier survival analysis of ZIPK expression in total 67 gastric cancer patients. High expression of ZIPK in primary tumors was significantly associated with better disease specific survival rates of GC patients (upper left panel); increased ZIPK expression only in metastatic lymph nodes was correlated with poorer disease specific survival (low left panel). ZIPK-HE, ZIPK high expression; ZIPK-LE, ZIPK low expression; ZIPK-IE, ZIPK increased expression; ZIPK-NIE, ZIPK non increased expression.

The above findings are in contrast with our previous study that showed that ZIPK is a tumor suppressor in human primary gastric cancers. Of note, BGC-823 and SNU-1 cell lines used here have been reported to display poor differentiation and aggressive biological behaviors [19–21]. Additionally, SGC-7901 cell line originated from metastatic lymph node, so we presumed that the oncogenic function of ZIPK would present at late stages when it could promote widespread metastases. To validate our hypothesis, the expression of ZIPK was examined in 79 pairs of primary gastric cancers and their matched metastatic lymph nodes by IHC. ZIPK expression could be evaluated informatively in 67 of 79 gastric cancers. The results showed that high expression of ZIPK was detected in 15/67 (22.4%) primary GCs and 28/67(41.7%) metastatic lymph nodes respectively (*p* = 0.016). In 12 out of 67 cases, ZIPK was high expressed in both primary and metastatic lesions and increased expression of ZIPK in metastatic tumors was found in 2 cases (Figure 7A). 16 cases that expressed ZIPK at low levels in primary tumors exhibited high expression only in lymph node metastases (Figure 7A). These findings led us to assess a possible association between ZIPK expression and clinicopathological features in primary tumors and their corresponding metastatic lymph nodes. High expression of ZIPK in primary or metastatic tumors was not significantly associated with age, gender, tumor histological type, TNM stage, and organ metastasis (Table 1). Interestingly, increased expression of ZIPK in lymph node metastases as compared to their primary tumors was significantly associated with stage IV (*p* = 0.012) and abdominal organ invasion (*p* = 0.005, Table 1). Furthermore, Kaplan–Meier analysis revealed that patients with high ZIPK expression in primary tumors had more favorable disease-specific survival than those with low ZIPK expression (Log-rank test, *P* = 0.03) (Figure 7B). However, patients with increased ZIPK expression only in metastatic lymph nodes had poor disease-specific survival (Figure 7B). Taken together, these results suggest that ZIPK may play a dual role in gastric cancer development and progression.

**Table 1:** Clinical association of ZIPK expression in primary gastric cancers and the matched metastatic lymph nodes

Variable	ZIPK protein						
	All case	High expression in primary tumor	*P*	High expression in metastatic lymph node	*P*	Increased expression only in metastatic lymph node	*P*
Age (years)			0.377		0.289		0.660
≤ 56^1^	29	5		10		7	
> 56	38	10		18		11	
Sex							
Female	11	3	0.671	7	0.086	6	0.146
Male	56	12		21		12	
Histological type^2^			0.118		0.702		0.686
WA	14	6		6		2	
PA	39	8		18		12	
MA	8	0		2		2	
SRC	6	1		2		2	
Stage			0.451		0.098		0.012
III	39	10		13		6	
IV	28	5		15		12	
Organ metastasis			0.524		0.153		0.005
M0	47	12		17		8	
M1	20	3		11		10	

1 Mean age

2 Histological type: WA, well/moderately differentiated adenocarcinoma; PA, poorly differentiated adenocarcinoma; MA, mucinous adenocarcinoma; SRC, signet ring cell carcinoma; UC,undifferentiated

## DISCUSSION

Several lines of evidence indicate that ZIPK mediates a variety of cell processes including cell death, smooth muscle contraction, proliferation, inflammation and migration [22]. In the past few years, ZIPK has been characterized as a tumor suppressor in various tumors. In our previous study, ZIPK was down-regulated in 68.9% of human primary GCs and low expression of ZIPK was significantly associated with invasion, metastasis and poorer prognosis [8]. John et al [9]. reported that three nonsynonymous point mutations in ZIPK (T112M, D161N, and P216S), identified in cancer cells, decreased or abolished kinase activity which could abrogate the growth inhibitory roles of ZIPK and lead to *in vitro* cell proliferation. However, DAPK family kinases have been demonstrated to perform an essential survival function. DAPK1 promotes TSC2 phosphorylation resulting in TSC1-TSC2 complex dissociation and mTORC1 signaling activation [11]. ZIPK increases Wnt/β-catenin-mediated transcription and gene expression through its interaction with NLK. Knockdown of ZIPK in human colon carcinoma cells induces a reduction of Wnt/β-catenin signaling and cell growth [23]. Our current study showed that patients at stages III–IV with high ZIPK expression in primary tumors had more favorable disease-specific survival than those with low ZIPK expression. This result is in accordance with our previous finding from 172 patients at all stages [8]. On the other hand, we also found that the frequency of ZIPK high expression was increased in metastatic lymph nodes compared to primary tumors. Moreover, increased expression of ZIPK in lymph node metastases was significantly associated with stage IV, abdominal organ invasion and poorer prognosis. Furthermore, our functional studies demonstrated that ZIPK had strong tumorigenicity, with overexpression of ZIPK promoting cell growth, proliferation, migration, invasion and tumor formation in nude mice. The role of ZIPK in promoting tumor metastasis was further validated in nude mouse metastasis models. Based on our clinical and experimental observations, we surmise that ZIPK functions as a tumor suppressor in primary tumor development and as a pro-oncogenic factor to enhance distant metastasis. The complex roles in carcinogenesis may be due to the involvements of ZIPK in a broad range of biological activities. Pro-cell death properties of ZIPK could contribute to tumor suppression via increasing tumor cell sensitivity to programmed cell death and maintaining genomic stability [22, 24, 25]. In the past few years, a series of experiments showed that the DAPK family trigger the autophagy process and strongly promote autophagic cell death [24]. ZIPK is involved in myosin II activation and the movement of Atg9-mediated complex during starvation-induced autophagy [6]. We have recently found that ZIPK inhibits cell proliferation, growth, migration and invasion through inducing autophagy in Ges-1 and MKN28 cells (unpublished data). In addition, ZIPK positively mediates platelet-derived growth factor-BB-induced vascular smooth muscle cell proliferation and migration [22]. Our current data confirm the effects of ZIPK on proliferation and migration in BGC-823, SGC-7901 and SNU-1 cells. These pro-oncogenic capabilities could promote cancer cells from lymph nodes to disseminate to distant organs. The alternative possibility would be that ZIPK plays different roles depending on the specific genetic and/or epigenetic alteration that occur during tumor progression. Accumulating evidences suggest that genetic or epigenetic divergence between primary tumor and metastasis results from the clonal evolution shaped by microenvironment-specific selection forces [26]. Our study have not deciphered the mechanism by which ZIPK can switch from a tumor suppressor to a pro-metastatic factor during the course of multi-step tumor progression. Thus, further investigations will be needed.

Metastasis is a multistep process including local invasion, intravasation, transport, extravasation, and colonization by which tumor cells disseminate from their primary site and form secondary tumors at a distant site. This process is essentially dependent on the prominent biological event referred to as epithelial-mesenchymal transition (EMT) [27, 28]. EMT which enables cells of epithelial phenotype to generate mesenchymal derivatives can be defined by the loss of cell–cell adhesion, the modification of cell morphology and the ability of cells to migrate [29–31]. In our study, ZIPK indeed induced EMT with increasing expression of β-catenin, mesenchymal markers, Snail and Slug. AKT is considered to be a key mediator of EMT induction in human epithelial malignancies. It is well accepted that AKT induces inactivation (phosphorylation) of GSK-3β, subsequently phosphorylating Snail, inducing its degradation and nuclear export, and ultimately triggering cell EMT [15]. Alternatively, AKT can directly phosphorylate IKKa, a component of IKK (IκB kinase) complex [16]. Phosphorylated IKKa can phosphorylate IκBa, leading to its ubiquitination and degradation by the proteasome, which can liberate NF-κB to enter the nucleus and up-regulate Snail and Slug expression to promote EMT [16–18, 32, 33]. In our study, over-expression of ZIPK induced AKT activation, and enhanced phosphorylation of IKKa, IkBa and NF-κB, suggesting that ZIPK regulates AKT/IκB/NF-κB signaling. Slug and snail were up-regulated by NF-κB activation, but expression of Slug was dramatically elevated in our experiments. A similar finding has also been reported in Hec251 cells [18]. Moreover, inhibition of AKT activation by LY294002 strongly suppressed expression levels of phosphorylated IKKa, phosphorylated IkBa, phosphorylated NF-κB, Snail and Slug, and prevented ZIPK-induced cell migration and invasion. Therefore, ZIPK promotes EMT and tumor metastasis through activating the AKT/IκBa/NF-κB signaling pathway. Here, we found that ZIPK promoted cell growth, proliferation and tumor formation *in vivo* and *in vitro*. The activation of EMT programs has been associated with the acquisition of stem cell traits by various cancer cells [34–36]. We detected the effect of ZIPK on stemness-associated genes and CSC surface markers in ZIPK- and empty vector-transfected BGC-823 cells. The results indicated that ZIPK could enhance stemness of gastric cancer cells. Cancer stem cells display self-renewal potential and the ability to initiate new tumors which may be of critical importance for colonization in the last step of the metastatic cascade [37]. Despite Wnt/β-catenin signaling [23], ZIPK-induced AKT activation may play a critical role in tumor growth. As mentioned above, ZIPK could not only promote cancer cell translocation by EMT, but also facilitate metastatic cell growth and colonization in distant organs.

Our observations have potential implications for gastric cancer treatment. At early stages, restoration of ZIPK expression might be effective in preventing or delaying subsequent development of gastric cancer, but it would be unwise in terminal stage disease because of the risk of promoting metastasis. A recent report showed that ZIPK modulate inflammatory responses in cultured vascular smooth muscle cells through activation of JNK, p38, and AKT, as well as reactive oxygen species (ROS) production. Using a DAPK inhibitor could prevent activation of AKT and hypertrophy in isolated mesenteric arteries via suppressing cell proliferation in spontaneously hypertensive rats [14]. Thus, DAPK inhibiter may be a promising agent for the therapy of terminal gastric cancer. Recent advances in our understanding of transforming growth factor (TGF)-β in tumor progression have indicated that it acts as a tumor suppressor early and as a pro-metastatic factor in late-stages. Administration of anti-TGF-β therapies for metastatic cancers has brought promising results in the preclinical and clinical settings [38, 39]. These successes will provide substantial evidence to improve personalized cancer therapy in the future.

In summary, our studies have found that over-expression of ZIPK promotes cell growth, invasion, tumor formation and metastasis. ZIPK enhances AKT activity and induces EMT through AKT-IκBa-NF-κB signaling. Increased expression of ZIPK in human lymph node metastases is significantly associated with stage IV, abdominal organ invasion and poor prognosis of GC patients. These findings reveal that ZIPK may serve as a pro-oncogenic factor for cancer cell translocation and colonization in distant organs.

## MATERIALS AND METHODS

### Clinical samples and cell lines

Paired tumor and metastatic lymph node specimens of 79 gastric cancer patients, who underwent gastrectomy between 2001 and 2003, were obtained from archives of the Department of Pathology of the First Affiliated Hospital, Sun Yat-sen University (Guangzhou, China). The patients included 66 males and 13 females, ranging in age from 27 to 84 years. Tumor histological types and differentiation were determined based on World Health Organization and Japanese Research Society for GC criteria [40, 41]. Tumor staging was defined according to the tumor-node-metastasis (TNM) classification system from International Union Against Cancer (UICC) recommendation [42]. All patients received chemotherapy (fluorouracil, mitomycin, and adriamycin) after gastrectomy and were followed up. Clinical and pathological features of these patients were summarized in Supplementary Table 1. All human tissues were obtained with informed consent and approval from the Committees for Ethical Review of Research in the First Affiliated Hospital, Sun Yat-Sen University. BGC-823, SNU-1 and SGC-7901 cell lines was kindly provided by Professor Jie Chen from the Department of Gastroenterology, the First Affiliated Hospital of Sun Yat-sen University. RPMI-1640 medium with 10% fetal bovine serum was used in culture.

### Establishment of ZIPK overexpressed cell lines

The ZIPK gene expression vector (EX-M0353-Lv130) and the vector control (EX-NEG-Lv130) were obtained from GeneCopoeia (GeneCopoeia, MD). To produce infectious lentiviral particles, 293FT cells cultured on 10 cm dishes were cotransfected with target plasmids and the packaging plasmids in the Lenti-Pac HIV packaging mix (GeneCopoeia, MD). Viral supernatants were harvested after 2 day transfection, and then these supernatants were added in BGC823 culture. After infection, cells were selected in 3 μg/ml puromycin for 2 weeks.

### RNA interference

Small interfering RNA (siRNA) (50 pM) against ZIPK (Genepharma, Shanghai) was transfected into SGC-7901 and SNU-1 cells in 6-well plates using Lipofectamine 2000 Reagent (Invitrogen, NY) according to the manufacturer's instructions. At 48 hours after transfection, the effects of gene silencing were measured via western blot analysis, XTT, wound healing and Transwell. The siRNAs targeting ZIPK were as follows: ZIPK-1,5′-CUGGAACAUUCCUGGAUUAT-3′;ZIPK-2,5′GGAACGAGUU-CAAGAACAUTT-3′;ZIPK-3,5′-GCAUCGCACACUUUGACCUTT-3′. Control siRNA was obtained from Genepharma (nonsilencing, catalog No.141201).

### Quantitative reverse transcription-PCR

Total RNA was extracted from cultured cells using TRIzol^®^ reagent (Invitrogen, NY). Single-stranded cDNA was generated from total RNA, using M-MLV reverse transcriptase and oligo (dT) 12–18 primers (Takara, Japan). The real-time quantitative PCR reaction was performed with the SYBR green detection system (Bio SYBR Green Master Mix, Takara, Japan). GAPDH served as an endogenous control. All cycle threshold (ct) values were determined in real time using CFX96™ Real-Time PCR Detection (Bio-rad, CA). The primer pairs for each target gene is listed in Supplementary Table 2.

### Immunoblot analysis

Total cellular and nuclear proteins were extracted from 70–80% confluent cultured cells, and separated using standard SDS–PAGE and transferred onto PVDF membranes. Membranes were probed with different optimally diluted primary antibodies, followed by a colorimetric method for immunosignal visualization. Rabbit anti-ZIPK antibody was purchased from ProSci Incorporated (Prosci, Poway, CA). Anti-E-cadherin, -N-cadherin,-β-catenin,-Vimentin, -Snail, and -Slug antibodies were purchased as part of the EMT Antibody Sampler Kit (Cell Signalling Technology, MA). Rabbit anti-AKT, -p-AKT, -p-PTEN, and -p-GSK-3β antibodies were purchased as part of the Phospho-AKT Pathway Antibody Sampler Kit (Cell Signalling Technology, MA). Rabbit anti-IKKα,-p-IKKα,-IκBα,-p-IκBα,-NF-κB, and -p-NF-κB antibodies were purchased as part of the Phospho-NF-kB Pathway Antibody Sampler Kit (Cell Signalling Technology, MA).

### *In vitro* tumorigenic assays

Foci formation assay, and soft agar assay were performed as described previously [43]. For foci formation assay, 1 × 10^2^ cells were plated in separate wells of a 6-cm^2^ plate. Surviving colonies (> 50 cells per colony) were stained with crystal violet and counted after 3 weeks of culture. For soft agar assay, 5 × 10^3^ cells were grown in 0.4% bactoagar on a bottom layer of solidified 0.6% bactoagar in 6-cm^2^ plates. After 3 weeks, colonies consisted of more than 80 cells were counted. All data were expressed as the means ± S.E.M. Triplicate independent experiments were performed for each assay. For cell growth assay, 5 × 10^2^ BGC-823, 1 × 10^3^ SGC-7901 and 1 × 10^3^ SNU-1 cells were seeded into 96-well plate respectively and cell growth rate was detected using cell proliferation XTT kit (Dojindo, Japan) according to the manufacturer's instructions.

### *In vivo* tumor formation assays

Animal experiments were performed in compliance with the guidelines for the Welfare of Experimental Animals in Sun Yat-sen University. For *in vivo* tumorigenicity assay, 5 × 10^5^ ZIPK- and empty vector-transfected cells were subcutaneously injected into the left and right dorsal flank, respectively, of 4- to 5-week old nude mice (five mice per group). Tumor volume was measured weekly over a 4-week period (formula: Volume = 0.5 × Length × 2 × Width).

### *In vitro* migration and invasion assays

ZIPK-transfected, ZIPK-silenced and their respective control cells were cultured on 35 mm dish until confluent, then wounded with a 10 μl pipette tip. Migration photos were captured at 0 hr, 24 hr and 48 hr after scratching. Transwell invasion assay was performed using polyethylene terephthalate-based migration chambers and BD BioCoat Matrigel Invasion Chambers (Becton Dickinson Labware, USA) according to the manufacturer's instructions. The number of invades cells was quantified in 10 fields under a 10 × objective lens.

### *In vivo* metastasis assay

Briefly, 5 × 10^5^ cells transfected with empty vector or ZIPK were intravenously injected through the tail vein of 4- to 5-week-old nude mice (five mice per group). After 8 weeks, the mice were euthanized and the number of surface metastases per lung was determined under a dissecting microscope. The lungs were excised and embedded in paraffin, where by hematoxylin and eosin (H&E) staining was performed to confirm the presence of tumors.

### Flow cytometry

ZIPK-overexpressed and control cells were trypsinized, washed twice with PBS and immunostained for 30 min on ice with monoclonal antibodies against CD24 (PE-conjugated, BD Bosciences, CA), CD133 (PE-conjugated, Miltenyi Biotec, GMBH) and CD44 (FITC-conjugated, BD Bosciences, CA,). Labeled cells were analyzed using a flow cytometer (FACS Calibur, Becton Dickinson). Background signals were established using control cells incubated with isotype-specific IgGs.

### Tissue microarray and immunohistochemistry

The tissue microarray (TMA) blocks were constructed according to a previously described method [8]. Staining was performed on 5-μm paraffin sections. Sections were deparaffinized in xylenes and graded ethanol, and then rehydrated in PBS. Antigen retrieval was performed by microwaving for 10 min in 10 mM citrate buffer (pH 6.0). Endogenous peroxidase activity was blocked with 3% hydrogen peroxide for 20 min. The TMA sections were incubated with rabbit polyclonal antibody against ZIPK (Prosci, Poway, CA) at a dilution of 1:100 overnight at 4°C. After incubation with peroxidase–linked secondary antibody (Envision detection system, Dako, Glostrup, Denmark), TMA sections were counterstained with Mayer's hematoxylin.

Immunostaining of ZIPK was evaluated by a semi-quantitative scoring system, described previously [8]. Cytoplasmic expression of ZIPK with staining index ≥ 4 was defined as high expression and cytoplasmic expression of ZIPK with staining index < 4 was defined as low expression in the present study. Lost, unrepresentative samples, and samples with too few tumor cells (< 100 cells) were excluded from the data analysis. All IHC results were evaluated by 2 independent pathologists without any prior knowledge of the clinicopathologic data. With discrepant results of the same slide, both observers reviewed again to obtain a consensus.

### Statistical analysis

Statistical analysis was conducted using SPSS version 13.0 (Chicago, IL). Student's *t*-test was performed to evaluate the data from cell growth, foci formation, soft agar assays, tumor formation and metastasis in nude mice, wound healing, transwell, Western blot and qPCR assays. Chi-square test was used to analyze the correlation between ZIPK expression and clinicopathological variables. Cancer specific survival rates were estimated by Kaplan–Meier analysis. The value of *p* < 0.05 was considered statistically significant.

## SUPPLEMENTARY FIGURE AND TABLES


