# Supplementary material for: Zipper-interacting protein kinase promotes epithelial-mesenchymal transition, invasion and metastasis through AKT and NF-κB signaling and is associated with metastasis and poor prognosis in gastric cancer patients
Source: Oncotarget. 2015 Mar 21;6(10):8323–38. doi: 10.18632/oncotarget.3200 (PMC4480755; doi:10.18632/oncotarget.3200)
Supplement: Supplementary file 1 [file oncotarget-06-8323-s001.pdf]

SUPPLEMENTARY FIGURE AND TABLES

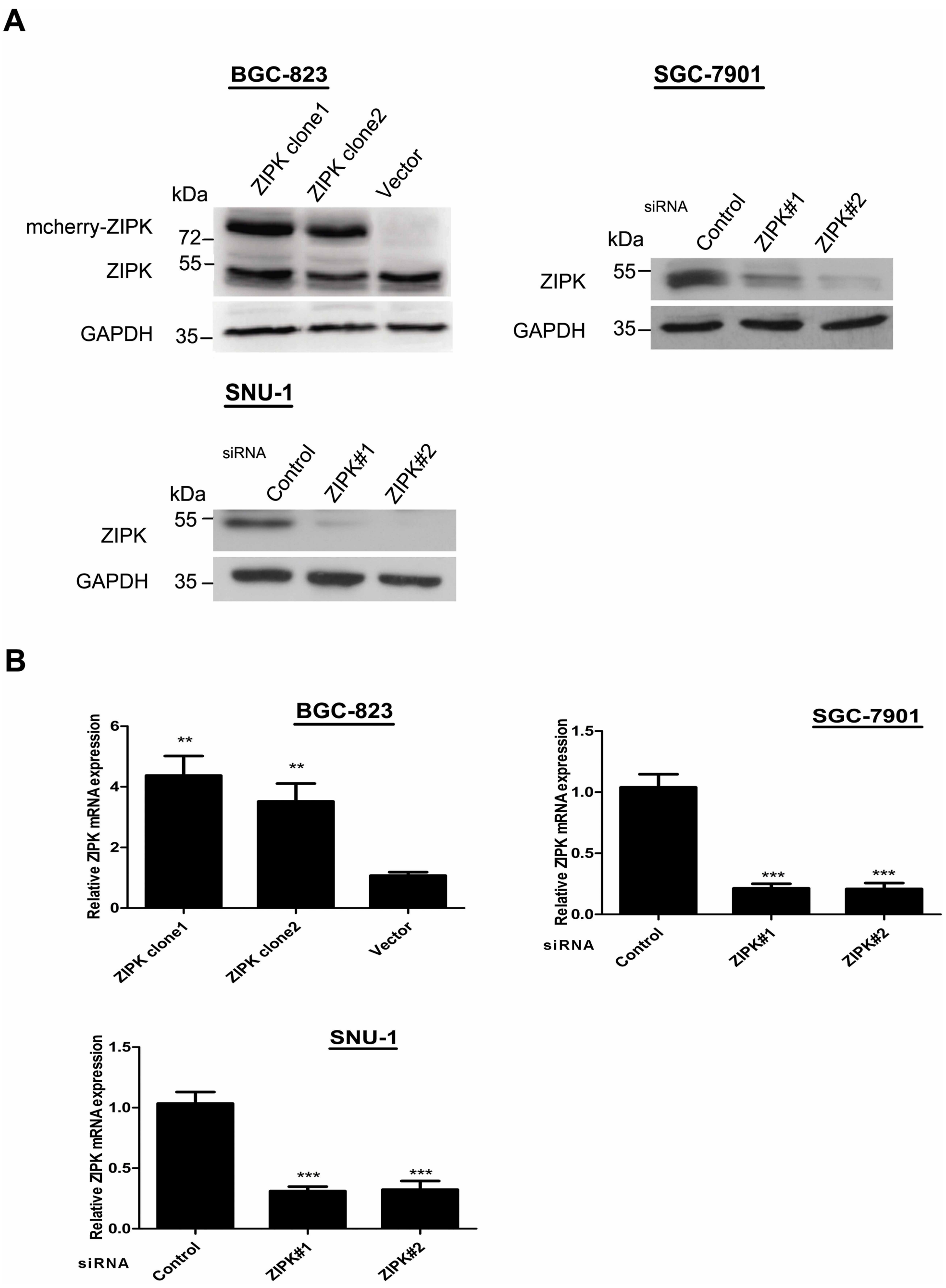

Supplementary Figure 1: (A) Stable ZIPK-overexpressing cell clones from BGC823 cell line were established. Expression of ZIPK was confirmed by Western blotting. The molecular weight of endogenous ZIPK was 52 kDa, and ectopic expression of mcherry -tagged ZIPK was 80 kDa. Western blotting demonstrated that expression of ZIPK was ablated in SGC-7901 and SNU-1 cells by siRNA. GAPDH was used as a loading control. **(B)** ZIPK mRNA level was detected in ZIPK-transfected, ZIPK-silenced and their respective control cells by qRT-PCR (**indicates $P < 0.01$, ***indicates $P < 0.001$, independent Student's *t*-test).

Supplementary Table 1: Staining index of ZIPK expression and patients' clinicopathological features in 67 informative cases

Case No.	Age	Sex	Histological type[1]	TNM stage	Organmetastasis	Survival Time (month)	Outcome[2]	Staining index of ZIPK expression	
								Primary tumor	lymph node
**1**	57	M	PA	IIIa	M0	101.0	L	4	4
**2**	60	M	WA	IIIa	M0	53.1	D	1	1
**3**	73	M	PA	IIIa	M0	19.3	D	1	1
**4**	59	M	WA	IIIa	M0	55.0	D	6	1
**5**	54	M	PA	IIIa	M0	8.8	D	1	1
**6**	73	M	PA	IIIa	M0	43.0	D	6	6
**7**	40	M	PA	IIIa	M0	22.4	D	2	2
**8**	59	M	MA	IIIa	M0	69.4	D	1	1
**9**	54	M	SRC	IIIa	M0	73.8	L	1	1
**10**	64	M	PA	IIIa	M0	40.0	M	6	1
**11**	54	M	PA	IIIa	M0	6.8	D	1	1
**12**	84	F	PA	IIIa	M0	71.2	L	6	6
**13**	61	M	PA	IIIb	M0	6.7	D	2	2
**14**	61	F	PA	IIIb	M0	4.5	D	1	1
**15**	61	M	PA	IIIb	M0	20.3	D	1	1
**16**	44	M	SRC	IIIb	M0	19.6	D	1	1
**17**	40	M	PA	IIIb	M0	59.0	D	1	1
**18**	69	M	WA	IIIb	M0	51.2	D	1	1
**19**	57	F	SRC	IIIb	M0	32.6	D	4	6
**20**	37	M	PA	IIIb	M0	26.1	D	2	6
**21**	68	M	WA	IIIb	M0	26.6	D	4	4
**22**	49	M	PA	IIIb	M0	8.1	D	1	1
**23**	48	M	SRC	IIIb	M0	9.3	D	1	1
**24**	53	M	PA	IIIb	M0	79.3	D	6	6
**25**	66	F	PA	IIIb	M0	97.5	L	1	6
**26**	58	F	PA	IIIb	M0	9.4	D	1	4
**27**	62	M	PA	IIIb	M0	23.1	D	1	6
**28**	62	M	PA	IIIb	M0	10.3	D	4	4
**29**	71	M	PA	IIIb	M0	88.5	M	1	1
**30**	58	M	WA	IIIb	M0	18.2	D	4	4
**31**	64	M	MA	IIIb	M0	21.2	D	1	1
**32**	48	M	PA	IIIb	M0	5.1	D	1	2
**33**	51	M	PA	IIIb	M0	49.9	D	1	6

(*Continued*)

Case No.	Age	Sex	Histological type[1]	TNM stage	Organmetastasis	Survival Time (month)	Outcome[2]	Staining index of ZIPK expression	
								Primary tumor	lymph node
**34**	51	M	PA	IIIb	M0	18.1	D	1	1
**35**	49	M	MA	IIIb	M0	81.0	L	1	1
**36**	19	F	PA	IIIb	M0	16.4	D	1	1
**37**	59	M	WA	IIIb	M0	47.4	D	1	1
**38**	49	M	WA	IIIb	M0	55.8	D	1	1
**39**	55	M	PA	IIIb	M0	11.5	D	1	1
**40**	43	M	PA	IV	M0	2.2	D	1	6
**41**	61	M	PA	IV	M1	18.1	D	1	4
**42**	46	M	PA	IV	M0	13.0	D	1	1
**43**	60	M	PA	IV	M1	11.1	D	1	1
**44**	40	M	PA	IV	M0	11.2	D	6	6
**45**	62	M	SRC	IV	M1	12.2	D	2	6
**46**	42	F	WA	IV	M1	41.1	D	1	1
**47**	62	M	MA	IV	M0	7.7	D	2	6
**48**	68	M	MA	IV	M1	13.1	D	1	1
**49**	64	F	MA	IV	M1	7.1	D	1	6
**50**	54	F	SRC	IV	M1	89.6	L	1	1
**51**	36	M	WA	IV	M1	10.7	D	2	6
**52**	70	M	PA	IV	M0	62.5	M	1	1
**53**	66	M	WA	IV	M1	10.9	M	4	2
**54**	68	M	MA	IV	M0	16.0	D	1	1
**55**	47	F	PA	IV	M1	6.7	D	6	9
**56**	64	M	PA	IV	M0	12.0	D	1	1
**57**	59	M	PA	IV	M1	7.6	D	1	4
**58**	68	M	MA	IV	M1	11.7	D	1	1
**59**	65	M	WA	IV	M1	7.5	D	1	4
**60**	70	M	WA	IV	M1	83.7	L	4	4
**61**	63	M	PA	IV	M0	10.4	D	1	4
**62**	46	M	PA	IV	M1	82.0	L	1	1
**63**	71	M	PA	IV	M1	11.5	D	1	6
**64**	35	M	WA	IV	M0	72.6	L	6	6
**65**	67	M	PA	IV	M1	7.3	D	1	1
**66**	39	F	PA	IV	M1	16.5	D	1	6
**67**	27	M	WA	IV	M1	6.2	D	1	1

[1]Histological type: WA, well/moderately differentiated adenocarcinoma; PA, poorly differentiated adenocarcinoma; MA, mucinous adenocarcinoma; SRC, signet ring cell carcinoma; UC, undifferentiated carcinoma
[2]Outcome: L, live D, death

Supplementary Table 2: Sequences of primers used in this study

Gene	Forward	Reverse
**ZIPK**	GAGGACCATTATGAGATGGGGG	CTCCCGCAGGATGTTCACC
**E-cadherin**	AAAGGCCCATTTCCTAAAAACCT	TGCGTTCTCTATCCAGAGGCT
**N-cadherin**	TTTGGGAGGGGTAAAAGTTC	AAGAAACAGGCCACCACCCTTT
**β-catenin**	CATCTACACAGTTTGATGCTGCT	GCAGTTTTGTCAGTTCAGGGA
**Vimentin**	GACGCCATCAACACCGAGTT	CTTTGTCGTTGGTTAGCTGGT
**Fibronectin**	CGGTGGCTGTCAGTCAAAG	AAACCTCGGCTTCCTCCATAA
**Snail**	TCGGAAGCCTAACTACAGCGA	AGATGAGCATTGGCAGCGAG
**Slug**	TGTGACAAGGAATATGTGAGCC	TGAGCCCTCAGATTTGACCTG
**AKT1**	TCCTCCTCAAGAATGATGGCA	GTGCGTTCGATGACAGTGGT
**GAPDH**	GCACCGTCAAGGCTGAGAAC	TGGTGAAGACGCCAGTGGA
